# Monolayer culture alters EGFR inhibitor response through abrogation of microRNA-mediated feedback regulation

**DOI:** 10.1038/s41598-024-56920-7

**Published:** 2024-03-27

**Authors:** Angela Florio, Sarah Johnson, Rebecca Salvatori, George Vasmatzis

**Affiliations:** 1 Biomarker Discovery Laboratory, Center for Individualized Medicine, Mayo Clinic, 200 First Street SW, Rochester, MN 55905 USA; 2 Mayo Clinic Graduate School of Biomedical Sciences, Rochester, MN USA; 3 Department of Molecular Medicine, Mayo Clinic, Rochester, MN USA; 4 Department of Laboratory Medicine and Pathology, Mayo Clinic, Rochester, MN USA

**Keywords:** Personalized medicine, Ex vivo drug screen, miR-146a, EGFR regulation, Lung adenocarcinoma, Patient-derived spheroids, Lung cancer, Biomarkers, Cancer models

## Abstract

Ex vivo drug screening is a potentially powerful tool for the future of cancer care, but the accuracy of results is contingent on the culture model. Both monolayer (2D) and spheroid (3D) culture systems offer advantages, but given the differences in mechanical environment, we hypothesized that that the suitability of one system over another would be critical for screening drugs with mechanical targets in mechanical tissues. HCC827 lung adenocarcinoma cells were challenged with EGFR tyrosine kinase inhibitors in monolayer and spheroid culture. RNA sequencing was performed on cells in both conditions to assess culture-induced transcriptional changes that could account for differences in drug response and differences in EGFR expression detected by immunostain. A microRNA microarray was performed to assess culture-induced differences in regulation of microRNA, and the impact of miR-146a-5p on drug response was verified by inhibition. Results were confirmed in human lung adenocarcinoma tissue. HCC827 spheroids were resistant to erlotinib and gefitinib, but significantly more sensitive in 2D culture. RNA-seq and immunostaining show a discrepancy in EGFR transcript and protein expression between the two conditions, which we attribute to miR-146a-5p. This microRNA targets EGFR and is differentially expressed between 2D and 3D culture. Inhibition of miR-146a-5p significantly increased erlotinib cytotoxicity, but validation in patient-derived spheroids suggests that the effect may be mutation-specific. Analysis of RNA-seq data suggests that cells in 2D culture become highly dependent on EGFR signaling to drive proliferation and cell spreading, resulting in a misleading level of sensitivity to EGFR TKIs, while the same cells in spheroid culture retain microRNA-driven EGFR feedback regulation that leaves them less vulnerable to EGFR inhibition. These findings underscore the need for close scrutiny of culture-induced effects on drug target regulation in model design for ex vivo drug screening.

## Introduction

The field of Individualized Medicine is moving toward ex vivo drug screening as a tool to enable cancer-agnostic treatment planning informed by genomic analysis of each patient’s tumor using tissue taken at biopsy or resection^1^. Evaluation of the level of cytotoxicity exerted on the patient’s own tumor cells by a selection of single and combination therapies identifies areas of sensitivity and resistance, allowing the clinician to optimize the therapeutic approach. Because these screens will be used to directly inform patient care, it is critical that the cell culture model recapitulate the tumor environment as closely as possible.

Culture models for drug screening on primary tissue often involve complex media formulations, extracellular matrix additives, specialized plates, and highly optimized protocols, but the fundamental decision is whether to culture the cells in monolayer or spheroid culture. Monolayer culture offers advantages in simplicity, streamlined handling, and ease of high-throughput adaptation for pre-clinical drug candidate screening; however, there are concerns that it does not mimic the tumor environment due to the mechanical response to a stiff plastic substrate, lack of three-dimensional cell–cell adhesions, lack of extracellular matrix, and upregulated proliferation, all of which result in changes in gene expression and signaling^2–9^. Spheroid cultures have been shown to mimic solid tumor architecture and retain the source tumor’s major genomic drivers^1^, but require specialized handling and present logistical barriers to high-throughput scaling. The question becomes: how well must the culture mimic the tumor to provide physiologically relevant drug screen results?

The question of the physical environment in culture becomes even more critical when targeting the epidermal growth factor receptor (EGFR). EGFR is over-expressed in 50 to 90% of non-small cell lung cancers (NSCLC) and carries activating mutations in 10 to 60% of cases depending on population^10–12^; these activating mutations can be up to 30-fold more catalytically active than wild-type EGFR^13^ and transduce signal even in the absence of ligand^14^. EGFR is targetable using tyrosine kinase inhibitors (TKIs) that bind to the active site and prevent downstream signaling, and first-generation TKIs are clinically approved for first-line treatment for lung cancers with known *EGFR* mutations^15,16^.

Study of EGFR in any capacity warrants close attention to the culture system. The differences between monolayer and spheroid culture are mechanical; monolayer culture forces cell spreading and planar polarity as cells adhere to the substrate, while spheroid culture allows cells to aggregate three-dimensionally and form cell–cell adhesions in all directions. EGFR is mechanoresponsive; it localizes to early focal adhesions^14^ and crosstalks with integrins in ways that can amplify or inhibit downstream signaling^17^, and it has also been shown to localize to cell–cell junctions and interact with E-cadherin^18^. While spheroid culture allows three-dimensional aggregation that mimics solid tumors in gross, lung adenocarcinoma arises from alveolar cells; histological examination shows that these tumors contain pseudostratified columnar cells arranged along a basal membrane surrounding the alveolar space (Supplementary Fig. S1). So, the question remains: which culture condition more closely recapitulates the tumor in terms of EGFR activity?

Here we investigate TKI response in monolayer and spheroid culture using EGFR-driven lung adenocarcinoma and describe how the choice of culture method can influence the physiological relevancy of drug screen results. HCC827 lung adenocarcinoma cells carry an *EGFR* exon 19 deletion that is the most common activating mutation^10^ and a marker of sensitivity to first-generation TKIs. We show how monolayer and spheroid culture produce significantly different drug screen results in these cells, and suggest that observable differences in proliferation are not the cause of this discrepancy, but rather a symptom of the differences in EGFR signaling taking place in these disparate environments. Through RNA-seq and microRNA microarray, we identify a possible feedback regulation mechanism that is disrupted in monolayer culture, resulting in misleading levels of drug sensitivity. Investigation using surgical lung adenocarcinoma tumor specimens supports the cell line data and illustrates the importance of model system design in drug screening applications.

## Results

### R1—HCC827 spheroids are resistant to EGFR TKIs and taxanes, but not cisplatin

**Figure 1:**
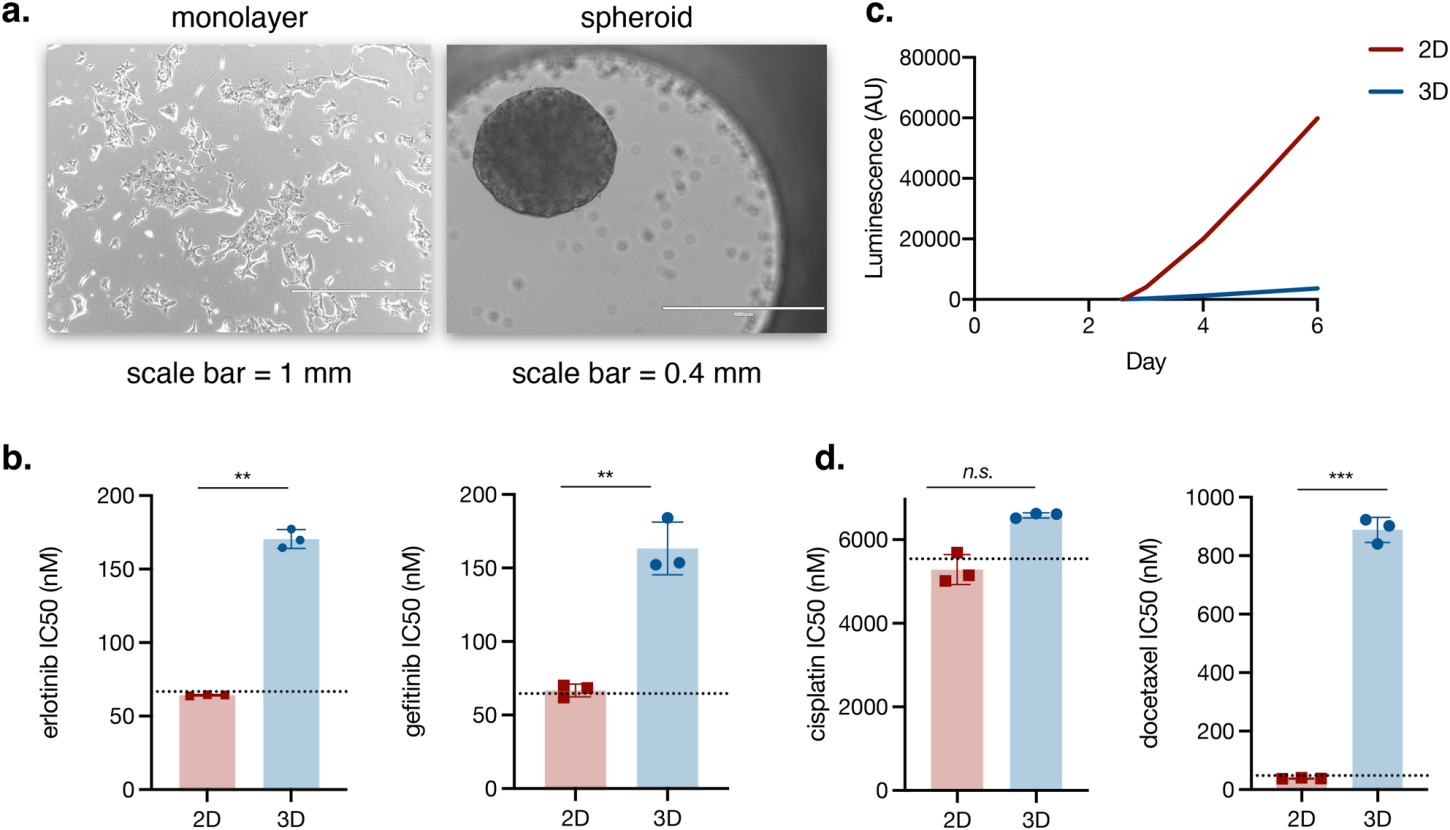
The effects of culture method on cells’ response to first-line lung adenocarcinoma therapies. (**a**) HCC827 cells were cultured for four days in either monolayer (2D) or hanging drop spheroid (3D) culture and challenged with erlotinib or gefitinib for 72 h. (**b**) The IC50 for cells in 2D culture aligns with that of the GDSC dataset while cells in 3D culture show increased resistance. (**c**) HCC827 cells were cultured in 2D and 3D for six days; ATP concentration was measured every 24 h. Cells in 2D culture begin proliferating earlier and show greater overall proliferation than cells in 3D culture. (**d**) HCC827 cells in 3D culture show increased resistance to the taxane docetaxel, but no significant difference in response to the platinum drug cisplatin. (**b**–**d**) Viability and ATP concentration were assessed via CellTiter Glo assay (Promega). (**b**, **d**). T-test with Welch’s correction; data represents the mean of three experiments; each experiment had six replicates per dose; error bars represent standard deviation. The dotted line indicates the IC50 for the indicated drug in HCC827 cells as published in the Genomics of Drug Sensitivity in Cancer (GDSC2) dataset^19^.

To investigate the influence of culture method on drug response, HCC827 *EGFR*-mutant lung adenocarcinoma cells were cultured as a monolayer (2D) or hanging drop spheroid (3D) for four days (Fig. 1a) and subsequently challenged with EGFR tyrosine kinase inhibitors (TKIs) erlotinib or gefitinib for 72 h. Post-treatment cell viability analysis shows that while cytotoxicity in 2D culture aligns with data published by the Genomics of Drug Sensitivity in Cancer (RRID:SCR_011956) database^19^, cells in 3D exhibit a significantly higher survival level that would read as resistant in a drug screen (Fig. 1b).

It is well-established that cells in 3D culture proliferate at a much lower rate than cells in 2D culture. We confirmed this and clarified the extent of the difference in HCC827 by measuring ATP concentration in 2D and 3D culture daily for six days; the resulting concentration curve shows that the 2D population begins expanding earlier than the 3D population and shows a much higher level of expansion overall (Fig. 1c). If the difference in proliferation is causing the difference in TKI response, it should be reflected in response to a drug that relies on proliferation to exert its cytotoxic effect; however, a 72-h challenge with DNA crosslinker cisplatin showed no significant difference in response between 2D and 3D culture (Fig. 1d). In a similar vein, we hypothesized that mechanical influences would be reflected in response to a drug with mechanical targets; after a 72-h challenge with docetaxel, which targets microtubules, cells in 3D culture survive at a significantly higher rate than cells in 2D (Fig. 1d) and would be read as resistant in a drug screen. Given EGFR’s mechanoresponsive nature, coupled with the difference in physical environment between the two conditions, we suspected that the disparity in cytotoxicity by EGFR TKIs may be due to differences in EGFR signaling induced by the mechanical environment.

### R2—HCC827 spheroids show an EGFR transcript/protein expression discrepancy

**Figure 2:**
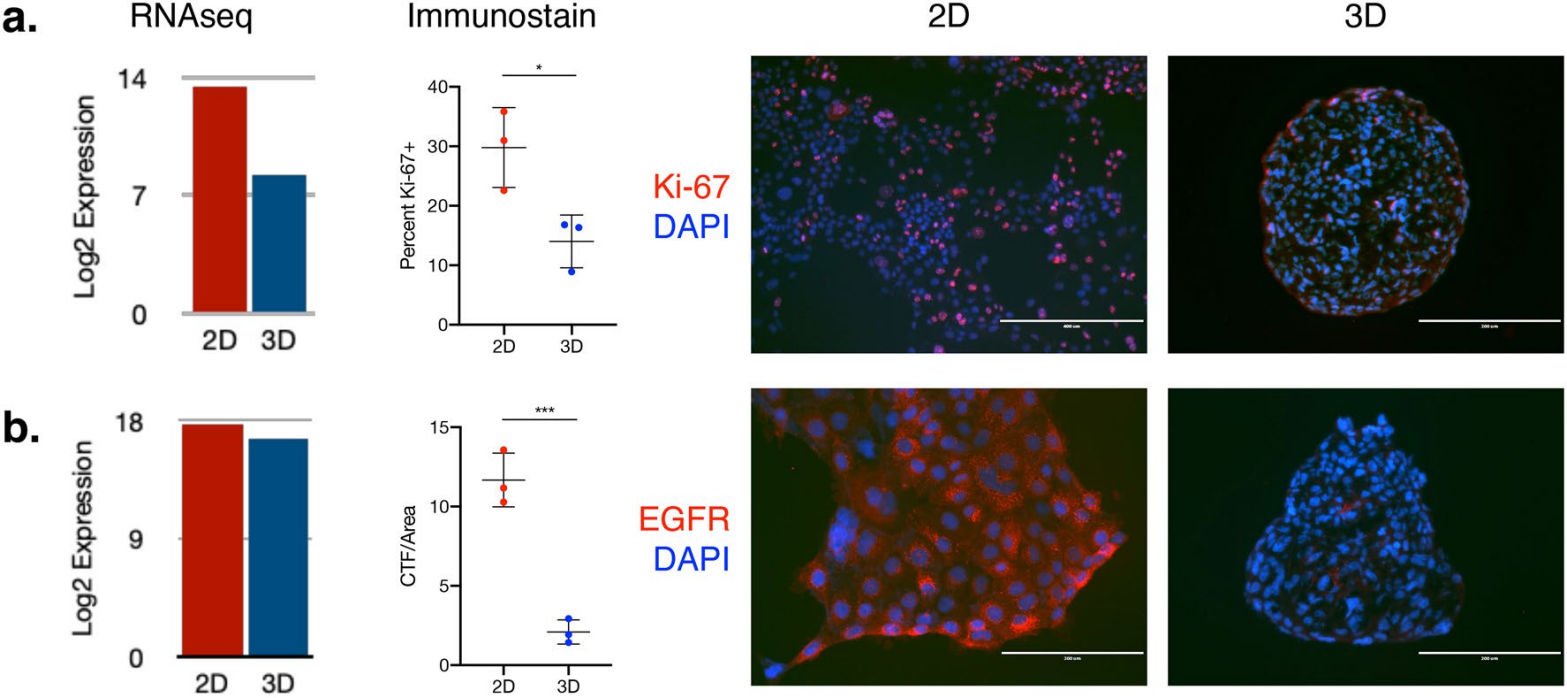
EGFR, but not MKI67, shows a discrepancy between relative transcript and relative protein expression. (**a**) HCC827 cells in 3D culture express lower levels of MKI67 as determined by RNA-seq (left); this is reflected at the protein level through immunostaining for Ki-67, which shows roughly 50% difference between the two conditions (middle, right). (**b**) EGFR transcript levels do not differ significantly between conditions (left), although immunostaining for EGFR shows significantly higher levels of EGFR protein in the 2D cells (middle, right). Student’s t-test, mean of three slides per condition. Error bars represent standard deviation.

To allow a more global analysis of transcriptional programs related to proliferation as well as EGFR signaling, we sequenced RNA collected from HCC827 cells in day four 2D and 3D culture. Genes involved in cell division show a clear 2D shift (Supplementary Fig. 4), reflecting the higher levels of proliferation seen in 2D culture. Immunostaining for cell division marker Ki-67 shows a significant difference in expression between cells in 2D and 3D, and that difference is proportionally reflected in expression levels of *MKI67* transcripts (Fig. 2a). However, while 2D and 3D culture show little difference in *EGFR* transcript expression, immunostaining shows significantly lower EGFR expression in 3D cells compared to 2D cells (Fig. 2b). This disconnect between transcript level and protein level may be explained by microRNA (miRNA)-mediated translation interference.

### R3—miR-146a-5p is up-regulated in 3D relative to 2D culture

**Figure 3:**
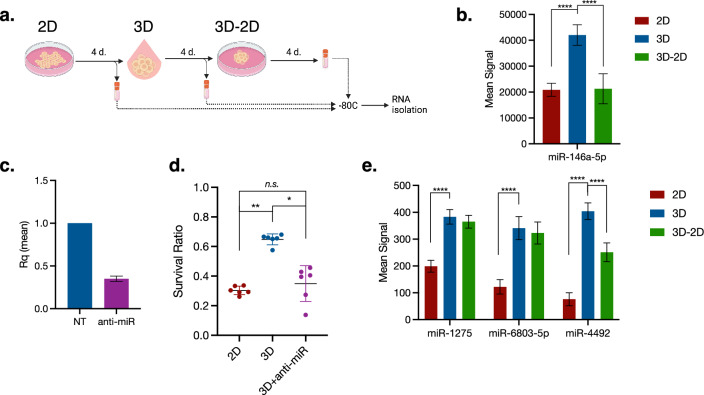
miRNA targeting EGFR is upregulated in spheroids and affects the response to EGFR TKI. (**a**) Cells were cultured in 2D for four days; half of the cells were then plated in 3D and the other half were stored at -80C. After four additional days, half of the spheroids were stored at -80C and the other half were returned to 2D culture for an additional four days and then stored at -80C. RNA isolation was performed in parallel. (**b**) miR-146a-5p shows a significant increase of expression in 3D culture, and a significant return to 2D levels in 3D-2D. (**c**). Transfection of spheroids with a miR-146a-5p inhibitor reduced expression by more than 50% compared to non-transfected cells. Expression measured by RT-qPCR, relative to non-transfected control. Error bars represent standard deviation. (**d**) Spheroids transfected with miR-146a-5p inhibitor show increased sensitivity to 150 nM erlotinib compared to non-transfected cells. Survival normalized to untreated controls; plot shows mean ± standard deviation. Kruskal–Wallis test with Dunn’s correction for multiple comparisons; n = 6. (**e**) Three additional miRNA that target EGFR show significant upregulation in 3D compared to 2D. (**b**, **e**). miRNA expression measured by miRNA microarray. Two-way ANOVA with Tukey’s test for multiple comparisons; n = 3; error bars represent standard deviation.

To identify the mature miRNA present in each condition, a miRNA microarray was performed on HCC827 cells in monolayer (2D) and spheroid (3D) culture, as well as spheroids that had been returned to monolayer culture (3D-2D) (Fig. 3a). Several miRNAs that show a pattern of change and recovery have putative targets in the EGFR-Cell Cycle axis^20–23^. miR-146a-5p shows a highly significant increase in 3D culture, with an equally significant decrease in 3D-2D culture (Fig. 3b), and was the most highly expressed miRNA in 3D culture. EGFR is a validated target of miR-146a-5p^24,25^, which also has high-quality putative targets^24^ (miRTarBase #MIRT004730; RRID:SCR_017355) in the MAPK^26–28^ (BIOCARTA M13863; RRID:SCR_006917), and PI3K-Akt^27–29^ (REACTOME M27162; RRID:SCR_003485) pathways, as well as Focal Adhesion^27,28,30^ (KEGG M7253; RRID:SCR_018145) and Adherens Junction^27,28,31,32^ (GO:CC M9849; RRID:SCR_002811) gene lists. From this data, we hypothesized that miR-146a-5p may be inducing TKI resistance in 3D culture by reducing EGFR protein availability.

### R4—miR-146a-5p expression influences cells’ response to EGFR TKIs

HCC827 cells in 3D culture transfected with a miR-146a-5p inhibitor and subsequently challenged with 150 nM erlotinib for 72 h showed an increase in sensitivity proportional to the decrease in miR-146a-5p expression (Fig. 3c,d). The level of erlotinib cytotoxicity upon miR-146a-5p inhibition still did not match that of cells in 2D culture, but as noted above, additional miRNA that target EGFR and points downstream were up-regulated in 3D culture, albeit expressed at lower levels (Fig. 3e). Taken together, the data thus far suggests that miR-146a-5p plays a role in cells’ response to EGFR TKIs and is regulated differently between 2 and 3D culture.

### R5—miR-146a-5p tissue validation

We next sought to validate the physiological relevance of these findings. We obtained surgical tissue samples of known EGFR-driven lung adenocarcinoma cases from the Mayo Clinic Thoracic Specimen Registry^33^ (NCT04118660); an aliquot of each sample was flash-frozen at the time of resection, and another aliquot was cryopreserved for subsequent culture. Because tumor recapitulation is the goal of ex vivo culture, data collected from the flash-frozen tissue was taken as the baseline for subsequent comparisons to the cryopreserved tissues in 2D and 3D culture.

**Figure 4:**
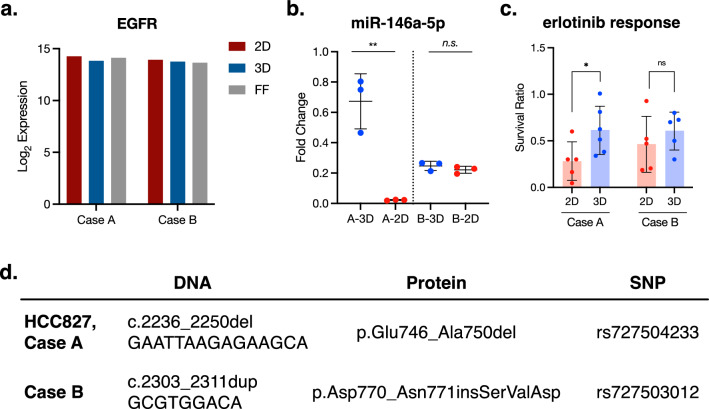
miR-146a-5p regulation and effect on drug response may be mutation-specific*.* (**a**) RNAseq shows that in both cases, EGFR transcripts are expressed at similar levels in both culture conditions and the tissue of origin. (**b**) Case A shows miR-146a-5p downregulation in 2D, reflecting the data collected from HCC827, while Case B shows no significant change. RT-qPCR; fold change relative to flash frozen control. Each point represents the mean of three technical replicates. Kruskal–Wallis test with Dunn’s correction for multiple comparisons. **p = 0.0045. (**c**) When challenged with 75 nM erlotinib for 72 h, Case A shows significantly higher resistance in 3D culture, which aligns with that seen in the HCC827 cells. Case B shows a nonsignificant difference in response. Each point represents a single well (2D) or spheroid (3D), and all spheroids of consistent size and shape were used. Kolmogorov–Smirnov test, *p* = 0.0476. (**d**) RNAseq shows that Case A carries the same EGFR exon 19 deletion as HCC827, while Case B carries an exon 20 insertion.

RNA was collected from the flash-frozen tissue as well as the dissociated cells cultured in 2D and 3D. While RNAseq shows that similar EGFR expression across all three conditions (Fig. 4a), RT-qPCR shows that miR-146a-5p expression is highest in the original tissue (Fig. 4b), suggesting that rather than 3D culture up-regulating expression, expression is actually down-regulated in 2D culture. It is important to note that while Case A shows a miR-146a-5p 2D/3D expression pattern reflecting that of HCC827, Case B shows little difference between 2D and 3D culture. When challenged with 75 nM erlotinib for 72 h, Case A showed significantly higher resistance in 3D culture compared to 2D culture (Fig. 4c), aligning with the response seen in HCC827. Case B showed a non-significant difference in response. The high variation in survival ratio is attributed to the different cell type ratios in the tumor-derived spheroid cultures. It is possible that the differences in miR-146a-5p regulation between these two cases are due to differences in the specific EGFR mutation. HCC827 and Case A carry the same *EGFR* exon 19 deletion, while Case B carries an *EGFR* exon 20 insertion (Fig. 4d).

### R6—2D and 3D cultures differentially direct transcription downstream of EGFR signaling

**Figure 5:**
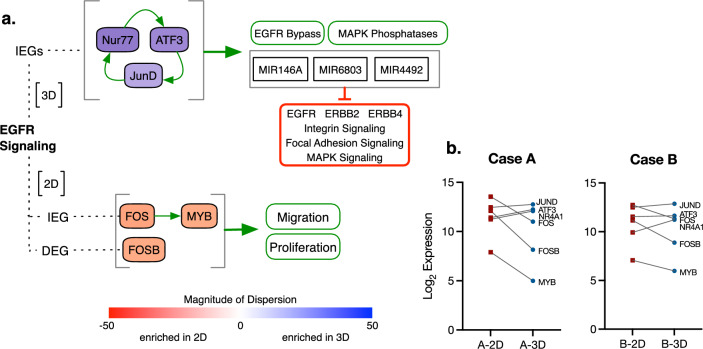
- RNA-seq data suggests a mechanism behind the differential regulation of miRNA targeting EGFR. (**a**) In 3D culture (top), EGFR signaling upregulates immediate early genes (IEGs) Nur77 and ATF3. Nur77 transcribes additional *ATF3*, and ATF3 transcribes *JUND*, which itself transcribes *NR4A1* (Nur77). This creates a self-sustaining loop that transcribes genes involved in EGFR bypass signaling, phosphatases that inactivate MAPK signaling, and miRNA that target EGFR signaling, integrin signaling, focal adhesion signaling, and MAPK signaling. In 2D culture (bottom), EGFR signaling upregulates immediate early gene *FOS*, which transcribes *MYB*, as well as delayed early gene (DEG) *FOSB*. Collectively, these genes transcribe over 400 genes involved in proliferation and migration as the cells spread and expand to fill available space. Genes are colored by magnitude of dispersion from 1:1 correlation; red indicates a shift toward 2D, blue indicates a shift toward 3D. (**b**) In Case A, expression of EGFR-induced transcription factors in 2D and 3D culture aligns with the changes seen in HCC827. Expression differences in Case B are less pronounced, and *FOS* is more highly expressed in 3D culture. See Supplementary Fig. 5 for correlation with flash-frozen tissue expression.

Integrating RNA-seq and miRNA microarray data allows us to propose a possible mechanism behind the difference in miR-146a-5p expression in 2D and 3D culture (Fig. 5a). RNA-seq expression data shows that transcription factors *ATF3*, *NR4A1*, *JUND*, *FOS*, *MYB*, and *FOSB* are expressed at significantly different levels between the two culture conditions (Fig. 5b, Supplementary Fig. 5), and all are triggered by EGFR downstream signaling^27,28^. In 3D culture, *ATF3* and *NR4A1* (Nur77) are induced as Immediate Early Genes (IEGs); *NR4A1* (Nur77) transcribes *ATF3*, ATF3 transcribes *JUND*, and JUND transcribes *NR4A1*; this creates a self-sustaining loop of transcriptional activation that collectively transcribes multiple miRNA that target EGFR and downstream signaling^21,23,34^. This loop also transcribes genes involved in EGFR bypass signaling as well as phosphatases that inactivate MAPK signaling, effectively up-regulating alternate routes for growth signal transduction and making the cell less reliant on EGFR itself; this may be a form of feedback regulation that reduces the amount of EGFR at the plasma membrane without completely shutting down growth factor signaling.

In 2D culture, EGFR signaling up-regulates FOS, which joins the AP-1 transcription factor complex and transcribes *MYB*, which itself is a transcription factor; FOSB is also up-regulated as a Delayed Early Gene (DEG). Together, these three transcription factors initiate transcription of over 400 genes involved in migration and proliferation^34^. The differences between 2D and 3D culture in expression of key EGFR-responsive transcription factors suggests that the morphological and behavioral differences between 2D and 3D culture are driven at least in part by EGFR signaling, in that 2D culture down-regulates inbuilt EGFR feedback regulation in order to drive cell spreading and proliferation. In the context of a drug screen, this mechanism increases TKI cytotoxicity due to the cells’ over-reliance on EGFR to drive the adaptation to 2D culture; however, the level of sensitivity suggested by 2D culture is not physiologically relevant, at least for EGFR TKIs.

## Discussion

Cells cultured as 3D models recapitulate the tumor architecture and environment^35^, retaining in vivo-like cell–cell interactions^36^, ECM production and deposition^35^, and major genomic drivers^1^; this results in more accurate representation of a tumor’s drug response^37,38^ despite patterns of hypoxia and drug penetration that may not always reflect that of the tumor^38–40^. While 2D culture has the advantage of convenience, evidence accumulated over the past several decades^6,41–45^ has shown that the mechanics of traditional monolayer culture bear comparatively little physiological relevance for drug screening applications^7–9^. This consideration becomes critical when screening drugs with mechanical targets, such as EGFR, as aberrant mechanical stimuli could result in alterations of target expression and activity^14^.

HCC827 cells have an *EGFR* exon 19 deletion that should make them suitable candidates for treatment with EGFR TKIs, and the GDSC IC50 studies^19^ show that they are among the most sensitive to these drugs of all cell lines tested. However, we found that when cultured in 3D systems, these cells show resistance to erlotinib and gefitinib; with the increasing push for drug screening models with greater physiologic relevancy, this data begs the question, “Which one is ‘correct’?”.

Our data shows that although 3D culture does indeed proliferate at a much lower rate than 2D culture, this difference does not impact cisplatin cytotoxicity. Cisplatin’s efficacy is reliant on replication, as the mechanism of action is to crosslink bases to create unrepairable DNA damage that arrests the cell cycle^46^. If the significant slowing of proliferation seen in 3D culture does not affect the response to such a replication-dependent drug, it is logical to explore alternative mechanisms. The difference in docetaxel response may be explained by the slowing of microtubule dynamics during spheroid aggregation and compaction^47^; it may be suggested that docetaxel’s microtubule stabilization enhances spheroid formation, as stable microtubules assist with the transport of adhesion molecules to the plasma membrane to reinforce cell–cell contacts^48^. This highlights the importance of the mechanical culture environment in the context of drug screening.

EGFR can be considered a mechanoresponsive receptor, as it crosstalks with integrins^17,49^ localizes to early focal adhesions and activates rigidity sensing on stiff substrates^14^ such as plastic. The mechanical differences between 2D and 3D culture have been widely studied. Traditional monolayer culture on plastic forces cells to spread out horizontally with no support for vertical spreading; this spreading a) increases the surface area in contact with the substrate, allowing the formation of a high number of focal adhesions, b) increases the surface area exposed to the drug-spiked media, and c) forces an apical-basal polarity that does not necessarily reflect that of tumor cells in situ and can alter signal transduction^7–9,50^.

Cells in 2D and 3D culture show similar levels of *EGFR* transcript expression as measured by RNA-seq, but significant differences in EGFR protein expression as measured by immunostaining. This discrepancy between transcript and protein expression may be explained by miRNA activity. Primary miRNA is transcribed into pre-miRNA, which is then processed by DICER and Drosha to form short mature miRNA fragments that can bind to target messenger RNA and inhibit translation. A variety of studies have identified miRNA that affect drug response through a variety of mechanisms, mostly by up-regulating or removing repression of EGFR bypass pathways^51,52^. We have identified miR-146a-5p as a correlative factor in EGFR TKI resistance and show that its expression is altered by the culture environment.

Due to the previously mentioned differences in proliferation, adherent cell lines are generally propagated in 2D culture. When detached from the plastic substrate and plated in a three-dimensional environment, the cells undergo a number of transcriptional alterations to adjust to the new environment including down-regulation of focal adhesions and up-regulation of cell–cell junctions. Our data shows that miR-146a-5p expression increases as cells move from 2D to 3D culture and begins to revert when 3D cells are returned to 2D culture, implying that miR-146a-5p expression is induced by the 3D environment. However, tissue samples show that miR-146a-5p expression is highest in tissue flash frozen at the time of resection, suggesting that miR-146a-5p is actually down-regulated in 2D culture. The two tissue samples show different expression patterns of miR-146a-5p; Case A shows miR-146a-5p down-regulation in 2D similar to that seen in HCC827 cells, while Case B shows no culture-induced adjustment. It is possible that the culture-induced differences in miR-146a-5p regulation and subsequent effects on TKI response may be specific to tumors with an *EGFR* exon 19 deletion or specific to tumors without an exon 20 insertion. A larger study with more samples that include the range of catalytically active EGFR mutations would be required to distinguish any mutation-specific effect from simple noise.

miR-146a-5p targets EGFR^53–55^ as well as heterodimerization partners ERBB2 and ERBB4^24,56^, which may increase the cell’s reliance on bypass pathways such as FGF/FGFR, HGF/c-MET, and PDGF/PDGFR. All of these ligand/receptor interactions transduce signals through the MAPK and PI3K/Akt pathways, thereby effecting EGFR-like outcomes in terms of survival, differentiation, growth, and proliferation even as EGFR itself is rendered inactive by TKIs. miR-146a-5p also targets the transcripts of focal adhesion proteins ROCK1^57,58^ and Rac1^24,59^, which play a key role in cytoskeleton regulation and cell polarity, as well as the transcripts of extracellular matrix proteins involved in lamellipodia formation; this could partially explain the difference in docetaxel response, as ROCK1 and Rac1 participate in microtubule dynamics. It is possible that miR-146a-5p is down-regulated in 2D culture to allow efficient focal adhesion formation and turnover, facilitating cell spreading.

Previously, miR-146a-5p had been classified as a tumor suppressor miR in non-small cell lung cancer^25^ by functionally inhibiting cell growth and migration, but our data shows that it may also contribute to TKI resistance by reducing EGFR availability and increasing reliance on bypass pathways. *MIR146A* is transcribed by Nur77 and JUND transcription factors, both of which are induced by MAPK signaling^30^. When EGFR is highly over-expressed, a high rate of receptor activation and internalization means that signaling through the MAPK pathway will proceed at a high rate. This signaling up-regulates MAPK-induced transcription, including that of *MIR146A*. *MIR146A* is then processed into mature miR-146a-5p, which subsequently degrades *EGFR* mRNA. This slows the re-supply of EGFR to the plasma membrane, forcing the cell to rely on other receptor tyrosine kinases to maintain incoming growth factor signaling. At this point, introducing an EGFR tyrosine kinase inhibitor will not induce the expected level of cytotoxicity because EGFR is no longer the primary receptor through which growth signaling is occurring.

## Conclusions

Together, the data shown here emphasizes the need for spheroid models in EGFR TKI screening and illustrates how drug screening requires careful consideration of the model system with close attention to both the drug’s target and mechanism of action. The role of miRNA in cancer and therapy is largely undefined due to the target-by-target, context-dependent functions of miRNA, the large number of validated human miRNAs, and the variable nature of their expression in vivo; however, growing interest in the field has already yielded actionable insights^57^. Further investigation will reveal the broader, system-wide implications of culture-induced effects on miR expression and subsequent cellular activity, with implications for both ex vivo and pre-clinical drug screening.

## Methods

### M1—cell and tissue culture

HCC827 cells (RRID:CVCL_2063; ATCC) were cultured in Dulbecco’s Modified Eagle’s Medium (DMEM) with 4.5 g/L glucose, L-glutamine, and sodium pyruvate (Corning #10-013-CV) with 10% fetal bovine serum (Corning #35-010-CV) and 1% Antibiotic/Antimycotic (Corning 15240-062) at 37C, 5% CO_2_ in either standard polystyrene tissue culture plates or Akura PLUS hanging drop plates (InSphero #CS-06-004-02). Spheroids were transferred to Akura 96 Spheroid Microplates (InSphero #CS-09-004-03) at day four for further experimentation.

Tissue samples were obtained from the Mayo Clinic Thoracic Specimen Registry^33^. Cryopreserved aliquots of each sample were dissociated using the GentleMacs Tumor Dissociation Kit (Miltenyi Biotech #130-095-929), and cultured in Advanced DMEM/F12 (Gibco #12634028) with 10% human AB serum (GeminiBio #100-512); 2% B-27 Plus Supplement (Thermo n#A3582801); 1% Glutamax (Gibco #35050079), 1 M HEPES (Sigma #H4034), and 1 M nicotinamide (Sigma #N3376); with antibiotics and growth factors present in trace amounts. Cells were plated in 2D or 3D as above. As spheroids take about 4 days to form, all cells in both conditions were cultured for 4 days before transfer to Akura 96 Spheroid Microplates for downstream experimentation to avoid confounding effects of different culture periods.

### M2—drug response

Cells were cultured for 4 days, then challenged with EGFR TKIs erlotinib (Chemie-Tek #CT-EL-002) or gefitinib (MedChemExpress #HY-50895), or taxane docetaxel (MedChemExpress #HY-B0011) in eight concentrations from 1 nM to 10 μM, or platinum drug cisplatin (MedChemExpress #HY-17394) from 1 nM to 100 μM for 3 days. Viability was assessed via CellTiter Glo Luminescent Cell Viability Assay (Promega #G7571), which uses luminescence to measure whole-cell ATP levels. Luminescence was measured using a Promega GloMax Multi-Detection System plate reader. Luminescence levels of treated wells were compared to untreated wells for quantification of relative viability.

### M3—ATP concentration curve

HCC827 cells were seeded in a 96-well plate or a 96-well Akura PLUS hanging drop plate. Three representative wells were assessed for viability via CellTiter Glo assay every 24 h for 6 days. Luminescence was measured using a Promega GloMax Multi-Detection System plate reader.

### M4—RNA sequencing

RNA was isolated using RNeasy Plus mini kit (Qiagen #74134) from day four cultures or macro-dissected flash-frozen tissue. Sequencing was performed by the Mayo Clinic Genome Analysis Core. Total RNA yield was determined by Qbit. Indexed RNA-seq library was generated using the TruSeq RNA Access Library Prep Kit (Illumina # RS-301-2001) and sequenced on a NovaSeq 6000 (Illumina, RRID:SCR_016387) platform. Raw sequencing data was analyzed using MAP-RSeq pipeline previously described^60^. Briefly, reads were aligned to the human genome build hg38 using STAR^61^ (RRID:SCR_004463), and FeatureCounts^62^ (RRID:SCR_012919) was used to generate gene and exon counts defined by Ensembl v78 (RRID:SCR_002344). The Subread package^63^ (RRID:SCR_009803) was used to quantify expression by RPKM. RPKM was normalized to a set of over 500 samples, and log_2_ transformed for deeper analysis.

### M5—immunostaining

For 2D samples, cells were cultured in chamber slides for 4 days before fixation with chilled 4% paraformaldehyde (PFA). For 3D samples, cells were cultured in hanging drop plates for 4 days, washed with phosphate buffered saline, fixed in 4% PFA, cryoprotected by incubation in 30% sucrose, and embedded in Tissue-Tek OCT (Sakura #4583) before cryosectioning at 5 μm. Sections that were folded, curled, or torn were discarded. All samples were blocked in bovine serum albumin, incubated overnight at 4C in primary antibody ([Cell Signaling Technology Ki-67 #9449 (RRID: AB_2797703)], [Santa Cruz Biotechnology EGFR (R-1) #sc-101 (RRID:AB_627494)]), incubated 1 h at room temperature in secondary antibody with conjugated fluorophore [Biotium #20115-1 (RRID:AB_10853942)], and mounted in VectaShield with DAPI [Vector Laboratories #H-1000 (RRID:AB_2336789)]. Images were acquired on an EVOS FL Color (Life Technologies) microscope. ImageJ was used to merge color channels (Supplementary Fig. S3) into a single RGB image.

Ki-67 staining was quantified as follows: DAPI images were used to obtain total cell count, and red channel images were used to manually count Ki-67+ cells. EGFR staining was quantified as follows: the cell mass was outlined on the DAPI slide, and the region of interest was transferred to the red channel image; area, mean, and integrated density were measured for the region of interest as well as three areas of background; Corrected Total Fluorescence was calculated as integrated density—(area × background mean) and divided by area. ImageJ (RRID:SCR_003070) was used for all image analysis. Intensity measurements were performed on original, unaltered images; some linear adjustments to brightness and contrast were made as needed for clarity on display images only.

### M6—miRNA microarray

HCC827 cells were cultured in monolayer for 4 days in DMEM (Gibco) 10% FBS, 1% penicillin/streptomycin. Half of the cells were stored at −80 C, and the other half were plated in hanging drop plates for four days. Half of the resulting spheroids were stored at −80 C, and the other half were plated onto flat-bottom 96-well tissue culture plates for four days. RNA was isolated using RNeasy Plus Mini Kit with protocol adjusted as per manufacturer’s instructions to preserve small RNAs. The microRNA microarray was performed by LC Sciences (Houston, TX; RRID:SCR_000140) using μParaflo™ chip technology with probes for all miRNA listed in Sanger miRBase Release 21 (mirBase.org, RRID:SCR_003152)).

### M7—miR-146a-5p quantification

miR-146a-5p was quantified from RNA via reverse transcriptase qPCR, using miRCURY LNA miRNA SYBR Green RT-PCR Kit (Qiagen #339345) with miR-146a-5p-specific primers (Qiagen #YP00204688) as per the manufacturer’s instructions. UniSp6 was used as an interplate spike-in calibrator, and miR-103a-3p was used as an endogenous control. qPCR was performed using QuantStudio 7 Pro System, software v2.6.

### M8—miR-146a-5p inhibition assay

miR-146a-5p was inhibited using the specific mirVana miRNA Inhibitor (Ambion #446084), delivered via Lipofectamine RNAiMAX Transfection Reagent (Invitrogen #13778-075) as per the manufacturer’s instructions. Cells were transfected for 48 h at 5 nmol inhibitor and 0.3 μL Lipofectamine in Opti-MEM (Gibco #31985062) before downstream experimentation.

### M9—statistical analysis

Unless otherwise specified, all statistical analysis was performed using Prism 9.0 [GraphPad (RRID:SCR_002798)]. IC50 data was interpolated from a dose–response curve generated using least-squares regression from luminescence data normalized to untreated controls (Supplementary Fig. S2). ATP curves were generated from luminescence data transformed by second-order smoothing. Specific tests for significance and their parameters are noted in figure legends.

### M10—ethics approval and consent to participate

Human tissue used in this study was obtained from the Mayo Clinic Thoracic Specimen Registry (Clinical Trial NCT04118660) from appropriately consented donors. Further ethical review was waived by the Mayo Clinic Internal Review Board due to the use of only banked tissue without patient data.

### Supplementary Information

Supplementary Information.

## Data Availability

Raw data for this study were generated at the Mayo Clinic Genome Analysis Core, and human sequence data generated in this study are not publicly available to preserve patient privacy. Derived data is available from the corresponding author upon reasonable request.

## Abbreviations

2D: Two-dimensional (monolayer) culture
3D: Three-dimensional (spheroid) culture
ATF3: Activating transcription factor 3
ATP: Adenosine triphosphate
DEG: Delayed early genes
EGFR: Epidermal growth factor receptor
FF: Flash-frozen tissue
FGF/FGFR: Fibroblast growth factor/receptor
FOS: Protein c-Fos
FOSB: FosB proto-oncogene, AP-1 transcription factor subunit
GDSC: Genomics of drug sensitivity in cancer
HGF/c-MET: Hepatocyte growth factor/receptor
IEGs: Immediate early genes
JUND: Transcription factor JunD
MAPK: Mitogen activated protein kinase
miR/miRNA: MicroRNA
MKI67/Ki-67: Marker of proliferation Ki-67
MYB: Transcriptional activator Myb
NR4A1/Nur77: Nuclear receptor subfamily 4 group A member 1
NSCLC: Non-small cell lung cancer
PDGF/PDGFR: Platelet-derived growth factor/receptor
PI3K-Akt: Phosphatidylinositol-4,5-bisphosphate 3-kinase—AKT serine/threonine kinase
Rac1: Rac family small GTPase 1
RNA-seq: RNA sequencing
ROCK1: Rho associated coiled-coil containing protein kinase
TKI: Tyrosine Kinase Inhibitor
