# Supplementary material for: Monolayer culture alters EGFR inhibitor response through abrogation of microRNA-mediated feedback regulation
Source: Sci Rep. 2024 Mar 27;14:7303. doi: 10.1038/s41598-024-56920-7 (PMC10973516; doi:10.1038/s41598-024-56920-7)
Supplement: Supplementary file 1 — Supplementary Information. [file 41598_2024_56920_MOESM1_ESM.pdf]

Monolayer culture alters EGFR inhibitor response through abrogation of microRNA-mediated feedback regulation

Angela Florio, Sarah Johnson, Rebecca Salvatori, George Vasmatzis

Supplementary Figures

Supplementary Fig. S1

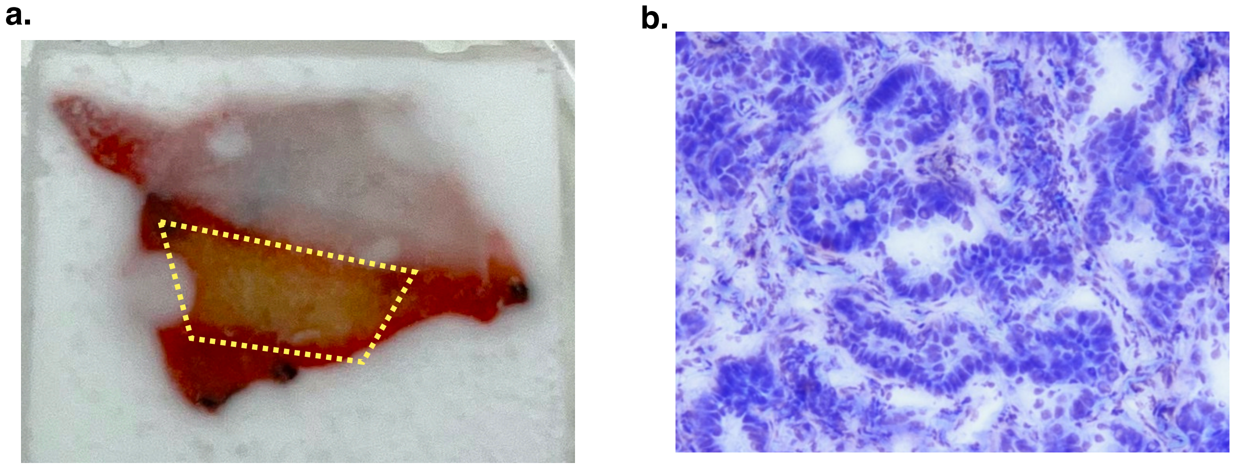

Lung adenocarcinoma macroscopic vs microscopic view. (a) In gross, the tumor reflects the three-dimensional aggregation of cells grown in 3D culture. The yellow dashed line delineates tumor from surrounding tissue. **(b)** Microscopic examination reveals glandular formations of pseudo-stratified columnar cells, suggesting the presence of a basement membrane-type common attachment substrate.

Supplementary Figure S2

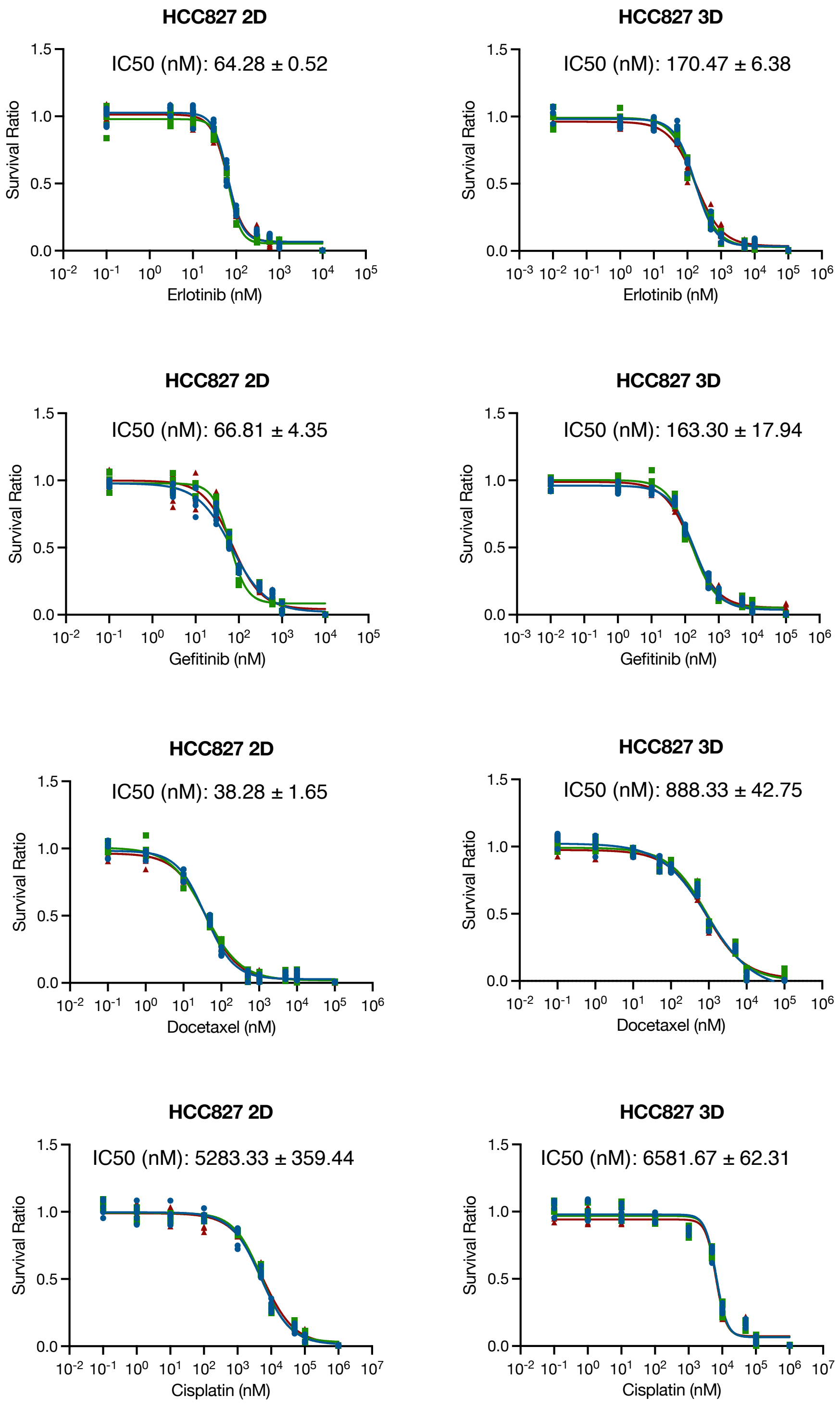

Dose response curves used to generate the IC50s in Figure 1. Each curve represents one experiment with six replicates at each concentration. See Methods section for statistics details.

Supplementary Fig. S3

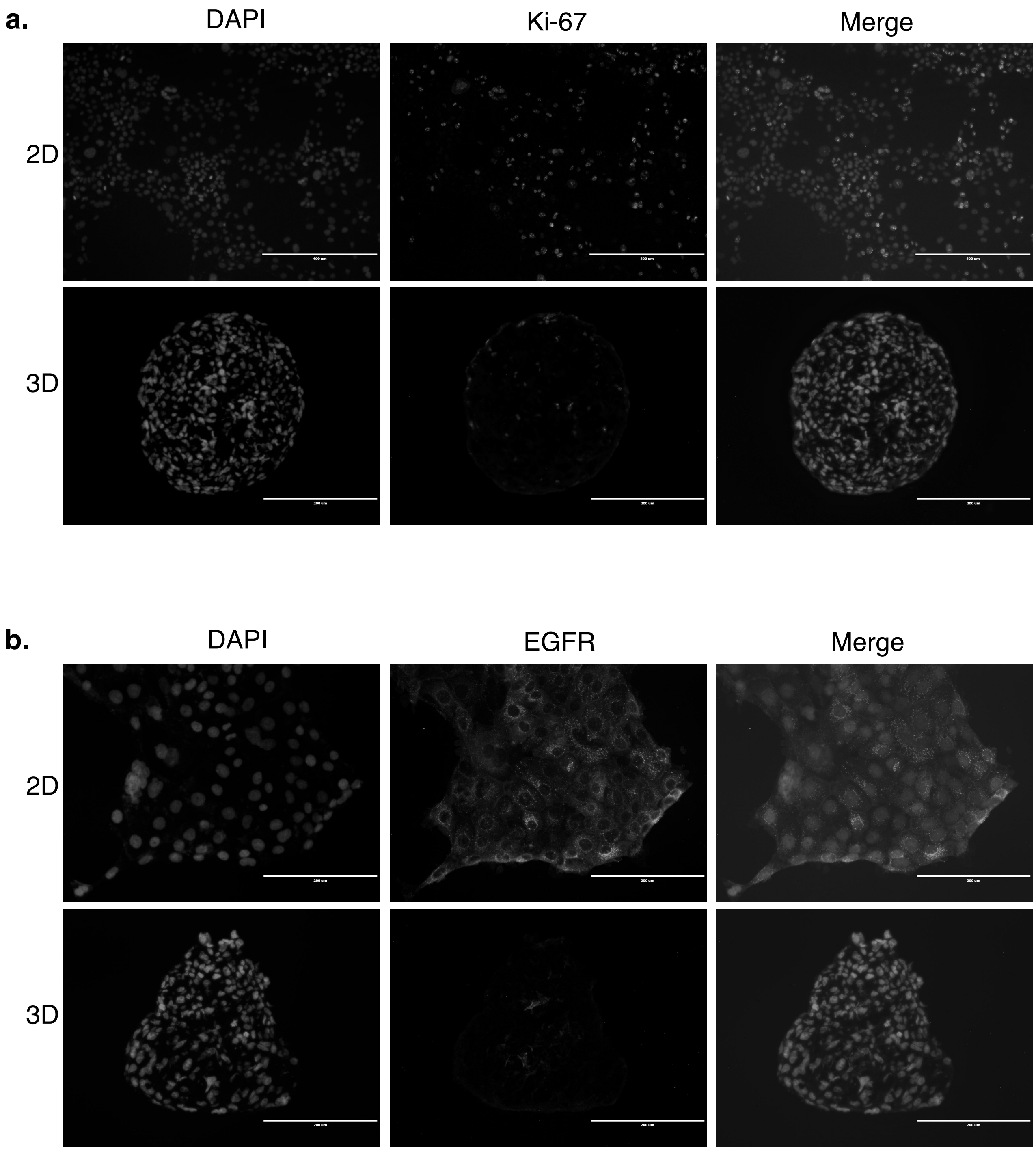

HCC827 immunostain individual channels. HCC827 cells were cultured as a monolayer (2D) or as hanging drop spheroids (3D) for four days. 2D cells were cultured in chambered slides. Spheroids were embedded in OCT and cryosectioned at 5 µm. The cells were immunostained for either Ki-67 **(a)** or EGFR **(b)**. ImageJ was used to merge color channels into an RGB image.

Supplementary Fig. S4

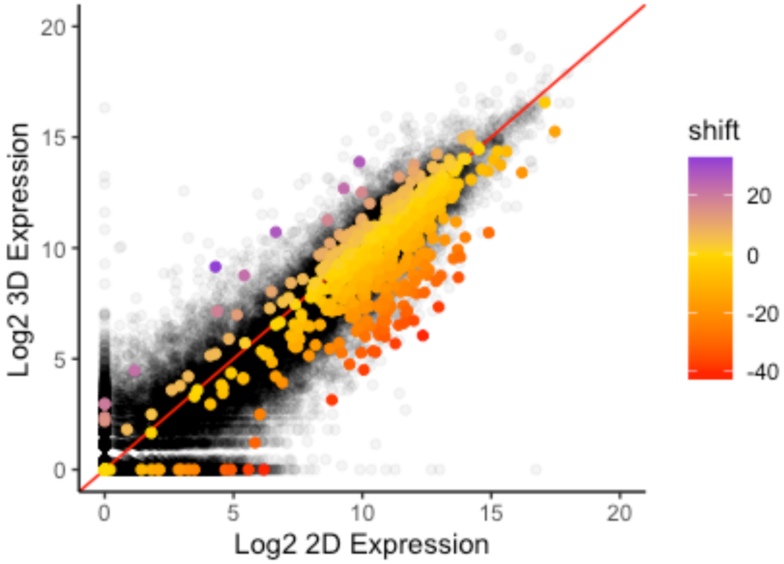

Cell division genes are differentially expressed in monolayer vs spheroid culture. HCC827 cells were cultured either as a monolayer or as spheroids for four days before RNA extraction. Monolayer expression values obtained by RNAseq were plotted on the x axis and spheroid expression values were plotted on the y axis to visualize the extent of correlation. Genes involved in cell division are highlighted, colored by magnitude of dispersion.

Supplementary Fig. S5

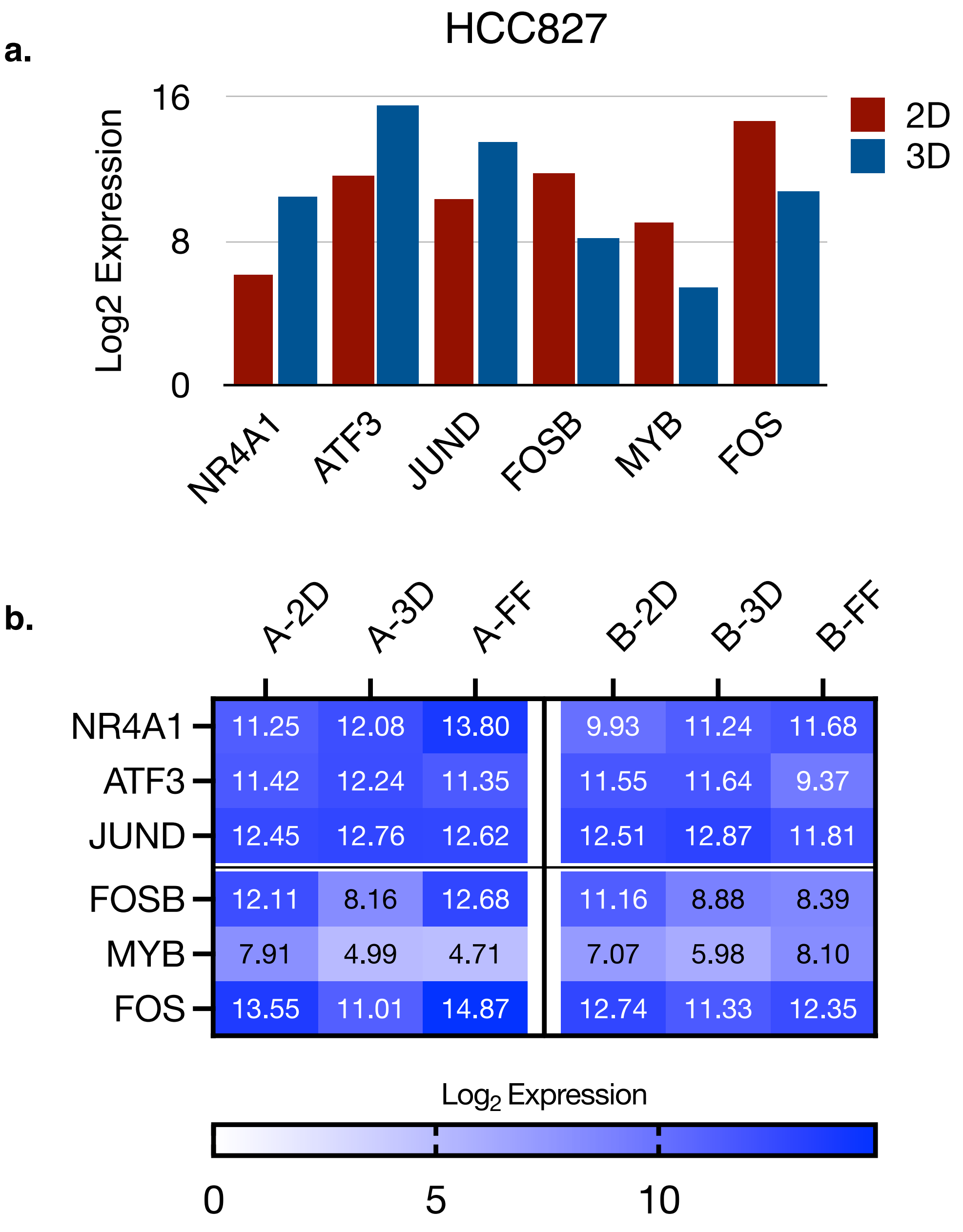

Transcription factors triggered by signaling downstream of EGFR are differentially expressed between 2D and 3D culture. **(a)** Expression levels in HCC827 2D and 3D culture. **(b)** Expression levels in Case A (left) and Case B (right) lung adenocarcinoma 2D and 3D culture as well as flash-frozen tissue (FF).
